# Relationship Between Morphofunctional Alterations of the Foot and Its Functionality in Patients with Fibromyalgia Syndrome: A Case–Control Study

**DOI:** 10.3390/jcm13216439

**Published:** 2024-10-27

**Authors:** María De Maya-Tobarra, Sara Zúnica-García, Alba Gracia-Sánchez, Esther Chicharro-Luna

**Affiliations:** 1 Department of Behavioural Sciences and Health, Nursing Area, Faculty of Medicine, Miguel Hernández University, 03550 Alicante, Spainagracia@umh.es (A.G.-S.); ec.luna@umh.es (E.C.-L.); 2 Institute of Health and Biomedical Research of Alicante (ISABIAL), 03010 Alicante, Spain

**Keywords:** fibromyalgia syndrome, foot function index, morphofunctional foot alterations, myofascial trigger points, foot pain

## Abstract

**Objective**: To evaluate the morphofunctional alterations in the foot and their association with functionality, considering aspects such as disability, pain, and limitations in daily activities in patients with fibromyalgia syndrome (FMS). **Methods**: A case–control study was conducted in patients with FMS (case group) and without FMS (control group), matched by age and sex. Foot posture was assessed using the foot posture index (FPI), along with the presence of hallux valgus (HV), trigger points, hyperkeratosis, and dorsiflexion of the first metatarsophalangeal joint and ankle. Foot functionality was evaluated using the foot function index (FFI) questionnaire. **Results**: A total of 100 women with FMS and 100 women without FMS, with a mean age of 61.97 ± 9.26 years, were recruited. HV (*p* < 0.001), hyperkeratosis (*p* < 0.001), pronated and supinated foot (*p* < 0.001), as well as limitations in dorsiflexion of the first metatarsophalangeal joint (*p* < 0.001) and the ankle with the knee flexed (*p* < 0.001) and extended (*p* < 0.001), along with the activity of the flexor hallucis brevis (*p* = 0.006), adductor hallucis (*p* = 0.006), and dorsal interosseous (*p* = 0.002) muscles, were significantly associated with the FFI, being higher in individuals with FMS, indicating greater impairment of foot functionality in these patients. Multivariate analysis revealed a statistical association between FMS and low educational level (OR = 2.57, 95% CI 1.05–5.72), the presence of another rheumatic disease (OR = 5.07, 95% CI 2.34–11), and the presence of any active trigger point (OR = 11.15, 95% CI 3.97–31.31). **Conclusions**: The study highlights the relationship between morphofunctional foot alterations, specifically the presence of active myofascial trigger points, and functionality in patients with FMS.

## 1. Introduction

Fibromyalgia syndrome (FMS) is a chronic pain syndrome characterized by widespread musculoskeletal pain, fatigue, and heightened sensitivity to tactile stimuli [1]. Its global prevalence is estimated to be around 2.10% [2], with 80% to 96% of those affected being women [3]. Although it commonly presents in middle age, it can affect individuals at any stage of life, including adolescents and the elderly [4].

The main symptom of FMS is chronic widespread musculoskeletal pain, which can manifest in various forms, such as spasms, stabbing pain, burning sensations, non-localized pain, tingling, hyperalgesia, and allodynia [3]. This persistent pain negatively impacts the quality of life, as well as causing cognitive and psychological impairment in affected individuals [5].

FMS is associated with several comorbid conditions, including sexual dysfunction, depression [6], migraine [7], and a high prevalence of psychiatric disorders, including sleep disorders, mood disorders, personality disorders, and anxiety [8]. Additionally, patients with FMS are at increased risk of mortality, including suicide, infections, and accidents. This syndrome significantly impacts health and society, leading to loss of work income before diagnosis, a low employment rate after diagnosis, and increased receipt of disability pensions. This results in an average cost of 27,193 EUR per patient per year [9].

Over 50% of individuals with this syndrome report foot pain [10] that negatively impacts their functionality [11], causing disability due to pain and symptom intensity [12], affecting activities of daily living [13].

Approximately 44.9% of patients use orthotic insoles as a supportive measure [12]. Additionally, foot-related alterations such as calluses, ingrown toenails [11], paresthesia, tarsal tunnel syndrome [14], and hypersensitivity to pressure in the plantar region have been reported in these patients [10].

FMS is a chronic disease characterized by widespread musculoskeletal pain and a variety of associated symptoms. However, few studies have focused on investigating the morphofunctional alterations at the foot level associated with FMS and how these may affect its functionality. The primary objective of this research was to evaluate the morphofunctional alterations of the foot and their association with functionality in patients with FMS (case group), compared to patients without FMS (control group). This comparison will provide a better understanding of the specific implications of FMS on foot morphology and function, providing a basis for the development of more effective and personalized therapeutic strategies.

## 2. Materials and Methods

### 2.1. Study Desing

A case–control study was conducted following the guidelines of the Strengthening the Reporting of Observational Studies in Epidemiology (STROBE) [15]. This study, carried out between March and May 2024, was approved by the Research Ethics Committee for Medicines (CEIm): PI2024-006.

### 2.2. Participants

Participants were recruited from various associations in the provinces of Alicante and Murcia, Spain. All participants were thoroughly informed about the study and signed informed consent forms, in accordance with the Declaration of Helsinki. The study was conducted by a podiatrist with over five years of clinical experience.

The case group consisted of individuals diagnosed with FMS, while the control group included individuals without FMS. Inclusion criteria for both groups were as follows: patients over 18 years of age, capable of walking at the time of examination, and willing to participate in the study by signing the informed consent. Patients with cognitive impairment, severe mental disorders, a history of lower limb surgery, or any musculoskeletal injury in the last six months were excluded from the study.

Both groups were matched for age and sex.

### 2.3. Sample Size Calculation

A sample size of 77 patients was estimated for the reference population, considering an FMS prevalence of 2.4%, a confidence level of 95%, and a margin of error of 10%, as well as accounting for a potential 10% loss.

### 2.4. Statistical Analysis

Statistical analysis of the study data was conducted using the Statistical Package of Social Sciences (SPSS^®^) v. 28. The qualitative variables are described by frequency distributions (counts and percentages), and the quantitative variables by means and standard deviations. Normality was assessed with the Kolmogorov–Smirnov test. Bivariate inferential analysis was conducted to identify associated risk factors, calculating the corresponding odds ratio and applying statistical tests such as chi-square or Fisher’s exact test for the qualitative variables, and Student’s *t*-test or the Mann–Whitney U test for quantitative variables, according to conditions of application. The age matching was done manually by preparing the data, sorting it by the age variable, and identifying subjects with similar ages to pair them. The Pearson correlation coefficient was used to assess the relationship between age and BMI, years of FMS progression, and onset of symptoms with the total FFI score. To identify the factors that are independently associated with foot functionality in patients with FMS, the adjusted odds ratio (OR) and confidence interval (CI) were obtained through a multivariate logistic regression analysis. All variables that showed a significant association in the bivariate analysis were included in the multivariate analysis, along with those variables that were considered relevant according to the scientific literature. Results were considered statistically significant when the *p*-value was <0.05.

### 2.5. Data Collection

Sociodemographic and clinical data were collected during the clinical interview. Family history of FMS, years of disease progression, age at diagnosis, and duration of symptoms were documented. Additionally, associated comorbidities and received pharmacological, adjuvant, and podiatric treatments were considered.

Foot type was assessed using the foot posture index (FPI) [16,17], which classifies the foot as pronated, supinated, or normal based on six postural criteria. A total score of 10 or more indicates a highly pronated foot, between +6 and +9 a pronated foot, between 0 and +5 a normal foot, between −4 and −1 a supinated foot, and −5 or less a highly supinated foot. The foot type was subsequently classified as normal, pronated, or supinated.

Hallux valgus (HV) was evaluated using the Manchester Scale. The scale classifies deformity into four grades: no deformity (grade 0), mild deformity (grade 1), moderate deformity (grade 2), and severe deformity (grade 3) [18,19]. A patient was considered to have HV if they obtained a grade of 1 to 3 on this scale.

The dorsiflexion of the first metatarsophalangeal joint was evaluated with the patient in the supine position using a goniometer. A limitation was considered if the movement was less than 65°.

The dorsiflexion of the ankle joint was evaluated with the patient in the supine position, ensuring a 90° angle at the ankle. A limitation was considered if the movement was less than 10°, evaluating both with the knee extended (to assess the triceps surae) and with the knee flexed (to assess the soleus muscle).

The location of hyperkeratosis on the metatarsal heads of both feet was analyzed to identify overload patterns.

A palpatory examination of specific muscles, including the dorsal interosseous, extensor digitorum brevis, quadratus plantae, flexor hallucis brevis, and adductor hallucis, was performed to identify trigger points related to FMS pain. A trigger point was considered active when the patient reported pain on palpation [10].

Foot functionality was assessed using the foot function index (FFI) questionnaire, a self-administered questionnaire consisting of 23 items divided into three subscales: pain (9 items), disability (9 items), and activity limitation (5 items). Each item is scored on a scale of 0 to 9. If an item is not applicable, it is left unanswered. The total score is calculated by summing the obtained scores, dividing by the maximum possible score of the answered items, and multiplying the result by 100. The final score, ranging from 0 to 100, reflects the impact on foot functionality; the higher the score, the greater the impact [20,21].

To evaluate the relationship between each morphofunctional alteration and foot functionality, an individual was considered to have an alteration when it was present in at least one of the two feet.

## 3. Results

A total of 200 women were recruited, including 100 patients with FMS (case group) and 100 individuals without FMS (control group), with a mean age of 61.97 ± 9.26 years. Table 1.

For those with FMS, the mean disease duration was 16.73 ± 9.59 years. The average age at disease onset was 45.68 ± 9.04 years, while the average time since symptom onset was 25.49 ± 13.39 years.

Several comorbid conditions were significantly more common in patients with FMS. Irritable bowel syndrome was more frequent in FMS patients (25% vs. 4%; *p* < 0.001), as was rheumatoid arthritis (RA) (11% vs. 3%; *p* = 0.027), osteoarthritis (67% vs. 19%; *p* < 0.001), migraine (33% vs. 3%; *p* < 0.001), and disc herniations (48% vs. 9%; *p* < 0.001). However, there were no significant differences between the groups in diabetes mellitus (5% in cases vs. 4% in controls; *p* = 0.733), dyslipidemia (40% in cases vs. 35% in controls; *p* = 0.465), hypertension (39% in cases vs. 27% in controls; *p* = 0.871), or hypothyroidism (18% in cases vs. 11% in controls; *p* = 0.160).

Various interventions were significantly more common among patients with FMS. Physiotherapy, psychological, psychiatric, and podiatric treatments were significantly more common in the FMS group. Additionally, patients with FMS had higher use of pharmacological treatments for pain, tranquilizers, antidepressants, and hypnotics compared to the control group.

Significant associations were found between the case group and the limitation of dorsiflexion of the first metatarsophalangeal joint and the limitation of dorsiflexion of the ankle with the knee extended in the left foot. Additionally, significant differences were observed in the presence of painful trigger points between cases and controls, with greater activation or pain in the case group for the flexor hallucis brevis, adductor hallucis, dorsal interosseous, extensor digitorum brevis, and quadratus plantae muscles. HV was significantly more prevalent in the right foot of the control group (81% vs. 68%). However, patients with FMS showed more advanced degrees of this deformity, both moderate and severe, bilaterally. Table 2.

The analysis of the FFI revealed significant differences between the case and control groups. Patients with FMS reported a higher average number of days with foot pain during the previous week compared to the control group (4.6 ± 2.49 vs. 1.90 ± 2.67 days; *p* < 0.001). They also had higher pain scores (63.43 ± 25.35 vs. 25.36 ± 52.02; *p* < 0.001), disability scores (59.18 ± 26.91 vs. 15.37 ± 21.96; *p* < 0.001), activity limitation scores (18.37 ± 21.95 vs. 1.90 ± 4.86; *p* < 0.001), and worse overall foot functionality (51.57 ± 22.13 vs. 14.52 ± 17.41; *p* < 0.001).

The items with the highest scores, indicating a greater degree of impact on each subscale, were as follows: pain level at the end of the day (6.48 ± 2.56 in cases vs. 2.24 ± 2.81 in controls; *p* < 0.001), difficulty standing on tiptoes (6.27 ± 2.73 in cases vs. 1.83 ± 2.73 in controls; *p* < 0.001), and activity limitation due to foot problems (3.39 ± 3.22 in cases vs. 0.51 ± 1.35 in controls; *p* < 0.001).

The duration of FMS did not show a correlation with the total FFI score (r = −0.156; *p* = 0.122) or with the years since symptom onset (r = −0.039; *p* = 0.701). Overweight or obesity, low educational level, smoking, sedentary lifestyle, having another rheumatic disease, and the use of plantar orthotics were significantly associated with the total FFI score (Table 3).

When relating morphofunctional alterations to the FFI, it was observed that the presence of HV, hyperkeratosis, pronated foot, supinated foot, limitation of dorsiflexion of the first metatarsophalangeal joint and of the ankle joint with the knee flexed and extended, and the activity of the flexor hallucis brevis, adductor hallucis, and dorsal interosseous muscles were significant, presenting a higher FFI in individuals with FMS (Table 4).

A share of 81% of patients with FMS had at least one active trigger point (flexor hallucis brevis, adductor hallucis, or dorsal interosseous muscles) compared to 19% of patients without FMS (*p* < 0.001).

Finally, multivariate analysis showed a statistical association between FMS diagnosis and low educational level (OR = 2.57, 95% CI 1.05–5.72; *p* = 0.021), having another rheumatic disease (OR = 5.07, 95% CI 2.34–11), and having any active trigger point (OR = 11.15, 95% CI 3.97–31.31; *p* < 0.001). The Nagelkerke R^2^ value was 0.51 (Table 5).

## 4. Discussion

The findings of this study reveal a complex relationship between FMS and various health and socioeconomic factors. Firstly, it was observed that patients with FMS had a higher prevalence of overweight and obesity compared to participants in the control group. Overweight and obesity are common in individuals with chronic pain due to various factors, such as increased biomechanical load, alterations in the gut microbiome, inflammation, and unhealthy lifestyle habits. Integrating weight reduction techniques into chronic pain treatment has proven to be more effective in reducing pain and disability than addressing these issues separately [22].

Patients with FMS exhibited a lower educational level, which is consistent with the findings of studies by Archenholtz et al. [23] and Bergström et al. [24], and a higher unemployment rate. This association could be explained by the functional and cognitive limitations that accompany FMS, negatively impacting the patients’ ability to maintain stable employment and pursue further education. These socioeconomic factors can exacerbate the symptoms and functional deterioration associated with FMS [25].

The presence of multiple comorbidities such as irritable bowel syndrome, RA, osteoarthritis, anxiety, depression, migraines, and disc herniations, which are significantly associated with FMS, suggests a complex and debilitating disease burden. Additionally, depression and anxiety are closely related to suicidal ideation in these patients [26].

Additionally, the high frequency of physiotherapeutic, psychological, psychiatric, and podiatric treatments, along with the use of pharmacological treatments for pain, psychotropic drugs, tranquilizers/anxiolytics, antidepressants, and hypnotics, reflects the need to address FMS from a comprehensive approach. This underscores the importance of personalized treatment that not only manages pain but also treats comorbidities and provides psychological and social support to the patient.

At the foot level, it was observed that patients with FMS had a significant limitation of dorsiflexion of the first metatarsophalangeal joint bilaterally, which could be related to the more advanced degrees of HV (moderate and severe) they experienced. Additionally, a study conducted by Keller et al. [27] confirmed the presence of elevated levels of joint stiffness in these patients, which aligns with the results obtained in our research. This degeneration of the first metatarsophalangeal joint could also be associated with the higher prevalence of other rheumatic diseases present in the studied population.

The results of this study revealed a significant association between active myofascial trigger points and FMS, particularly highlighting the more notable involvement of the dorsal interosseous muscles of both feet. The study conducted by Tornero-Caballero et al. [10] compared myofascial trigger points in women with FMS with and without foot pain, and found that women with foot pain and FMS showed greater pressure sensitivity in the plantar region, identifying the flexor hallucis brevis and adductor hallucis as the most common active myofascial trigger points. Additionally, topographic maps of pressure pain sensitivity revealed that women with FMS and foot pain had a lower pressure pain threshold, especially in the calcaneus bone. However, our findings indicate that the most affected muscles were the dorsal interosseous muscles.

Regarding foot functionality, it was observed that patients with FMS exhibited greater impairment compared to controls. This was evidenced by a significantly higher score on the FFI questionnaire (51.57 ± 22.13 vs. 14.52 ± 17.41; *p* < 0.001). The findings are consistent with the results of the study by Ciaffi et al. [11], which found that patients with FMS attending podiatry consultations showed greater foot functionality impairment compared to those without this disease (63.4 ± 23.0 vs. 53.2 ± 20.3; *p* < 0.001). Additionally, we found that the foot pain subscale was the most affected, followed by disability and limitation. These results also align with the findings of Ciaffi et al. [11] and López-Muñoz et al. [12].

The items with the highest scores in each FFI subscale, and therefore with the highest degree of impact, were pain at the end of the day, difficulty standing on tiptoes, and activity limitations due to foot problems. These results are consistent with those found by López-Muñoz et al. [12], where the highest mean scores related to foot pain were at the end of the day (7.80 ± 2.32) and when walking with shoes (7.51 ± 2.31). The greatest difficulties reported by the patients were standing on tiptoes (7.53 ± 2.70), walking quickly (7.35 ± 2.63), and climbing stairs (6.86 ± 2.59).

Regarding morphofunctional foot alterations and functionality, it was observed that active myofascial trigger points are associated with greater functional deterioration of the foot, especially in the flexor hallucis brevis, adductor hallucis, and dorsal interosseous muscles. These findings support previous theories suggesting an association between regional pain in FMS and the presence of active trigger points [28,29]. This could be explained by the nature of pain in FMS, which is not diffuse and generalized but concentrated in specific body areas [30]. It would be advisable for the exploration of trigger points to become a routine practice in evaluating the foot’s condition.

Appropriate treatment of foot pain could have significant clinical relevance for patients with FMS. In this study, as well as in previous research, it has been observed that this group of patients reports higher levels of disability, including gait pattern alterations [31,32]. It is recommended to undergo a personalized biomechanical assessment to determine whether a plantar orthosis is necessary to improve foot functionality and relieve pain [33]. Likewise, it is suggested to use footwear with an appropriate length, wide toe box, cushioned sole, and low heel to prevent overloading the forefoot. The use of toe separators may also help reduce pain [34]. Lastly, engaging in moderate-intensity resistance training could improve multidimensional functionality, pain, sensitivity, and muscle strength [35].

However, our study has several important limitations that should be considered when interpreting the results obtained. The primary limitation lies in the episodic nature of the disease, characterized by acute pain episodes. It was not evaluated whether, at the time of inclusion in the study, the patients were experiencing these flares, which could have influenced the questionnaire scores due to a temporary increase in the intensity and number of associated symptoms.

Another limitation is the inability to generalize the results to the male population, as the study included only women with FMS. However, it is relevant to note that this syndrome predominantly affects women, which partially justifies the sample choice.

To mitigate selection bias, the control group participants were matched by sex and age with those of the case group. This strategy was implemented to reduce the influence of confounding variables related to these characteristics. Nonetheless, despite this attempt to minimize bias, the lack of matching of cases and controls based on BMI, a significant variable between the groups, could have influenced the results obtained.

These considerations are essential for a careful and accurate interpretation of the study findings, highlighting the need for future research to address these limitations. Moreover, it would be beneficial to expand the sample size to generalize the results and develop specific tools or questionnaires to assess the foot in these patients. Additionally, it would be valuable to investigate how joint hypermobility in the foot, commonly seen in conditions such as Ehlers–Danlos syndrome, affects functionality in fibromyalgia patients. Since both syndromes share characteristics such as joint instability, hyperlaxity, and chronic pain, the combination of these conditions could exacerbate functional limitations and pain in these patients [36,37].

## 5. Conclusions

This study highlights a relationship between morphofunctional foot alterations, especially the presence of active myofascial trigger points, and functionality in patients with FMS. The identification of significant differences in joint mobility and muscle activation between FMS patients and healthy controls underscores the need for specific therapeutic approaches to address these functional limitations. Additionally, the higher prevalence of comorbidities in FMS patients suggests that these individuals require a multidisciplinary management approach that considers both physical aspects as well as socioeconomic and educational factors.

## Figures and Tables

**Table 1:** Sociodemographic and lifestyle characteristics in cases and controls.

Variables	Case Group (FMS) *n* = 100	Control Group (No FMS) *n* = 100	*p* Value
	Mean ± SD *n*	Mean ± SD *n*	
Age (years)	62.18 ± 9.21	61.76 ± 9.34	0.375 ^b^
BMI (kg/m^2^)			0.008 ^a^ *
Underweight (BMI < 18.5)	1	0	
Normal weight (BMI 18.5–24.9)	33	55	
Overweight (BMI 25–29.9)	40	32	
Obesity (BMI ≥ 30)	26	13	
BMI (kg/m^2^)	27.03 ± 4.99	25.18 ± 4.16	0.004 ^c^ *
Educational level			<0.001 ^a^ *
Without formal education	9	2	
Primary studies	47	18	
Secondary studies	33	33	
University studies	11	47	
Marital status			0.496 ^a^
Single	9	12	
Married	64	54	
Separated or divorced	17	19	
Widowed	10	15	
Family cohabitation			1 ^a^
Lives alone	26	26	
Lives with someone	74	74	
Employment status			<0.001 ^a^ *
Employed	21	43	
Unemployed	25	7	
On sick leave	11	6	
Retired	43	44	
Smoking habit			0.128 ^a^
Current smoker	11	17	
Ex-smoker	30	38	
Non-smoker	59	45	
Physical activity			0.606 ^a^
Sedentary lifestyle	23	20	
Physically active	77	80	
Family history of FMS			0.002 ^a^ *
Yes	27	10	
No	73	90	

Abbreviations: BMI, body mass index; FMS, fibromyalgia syndrome. ^a^ Chi-square test. ^b^ Student’s test. ^c^ Mann–Whitney U test. * Statistical significance *p* < 0.05.

**Table 2:** Relationship between the morphofunctional alterations of the right and left foot in the case group and control group.

Variables	Right Foot		*p* Value	Left Foot		*p* Value
	Case Group (FMS) *n* = 100	Control Group (No FMS) *n* = 100		Case Group (FMS) *n* = 100	Control Group (No FMS) *n* = 100	
	*n* Mean ± SD	*n* Mean ± SD		*n* Mean ± SD	*n* Mean ± SD	
Type of foot			0.323			0.475
Normal	77	82		82	83	
Pronated	17	17		14	10	
Supinated	6	8		4	7	
HV	68	81	0.035 *	73	79	0.321
HV			0.002 *			<0.001 *
No deformity (grade 0)	32	19		27	21	
Mild deformity (grade 1)	37	62		37	64	
Moderate deformity (grade 2)	27	19		33	14	
Severe deformity (grade 3)	4	0		3	1	
Hyperkeratosis	85	82	0.568	83	84	0.849
Limited DF of 1st MTP joint	100	98	0.155	100	100	-
DF 1st MTP joint mobility	31.45 ± 12.08	38.11 ± 13.17	0.004 *	33.37 ± 13.38	38.23 ± 11.54	0.010 *
Limited DF of TPT joint (with knee flexed)	17	13	0.428	12	17	0.315
DF of TPT joint mobility (with knee flexed)	14.15 ± 5.42	14.58 ± 4.80	0.679	14.23 ± 5.61	14.86 ± 5.53	0.244
Limited DF of TPT joint (with knee extended)	52	51	0.887	63	47	0.023 *
DF of TPT joint mobility (with knee extended)	8.72 ± 4.72	8.43 ± 3.24	0.915	8.21 ± 4.15	8.96 ± 3.87	0.062
Flexor hallucis brevis active	43	3	<0.001 *	48	8	<0.001 *
Adductor hallucis active	43	3	<0.001 *	48	8	<0.001 *
Dorsal interosseous active	52	10	<0.001 *	52	11	<0.001 *
Extensor digitorum brevis active	26	0	<0.001 *	23	1	<0.001 *
Quadratus plantae active	40	1	<0.001 *	43	4	<0.001 *

Abbreviations: 1st MTP, first metatarsophalangeal; DF, dorsiflexion; FMS, fibromyalgia syndrome; HV, hallux valgus; TPT, tibiofibular-talar. Chi-square test. * Statistical significance *p* < 0.05.

**Table 3:** Relationship between sociodemographic variables and the FFI in cases and controls.

Variables	Total FFI Score		
	Case Group (FMS)	Control Group (No FMS)	*p* Value
	Mean ± SD	Mean ± SD	
Overweight or obesity (BMI ≥ 25 kg/m^2^) (*n* = 112)	52.14 ± 20.75	19.99 ± 19.01	<0.001 *
Low educational attainment (no formal education or only primary education) (*n* = 76)	52.14 ± 20.75	20.75 ± 19.01	<0.001 *
Not actively employed (*n* = 136)	54.53 ± 20.90	17.69 ± 18.89	0.058
Current smoker (*n* = 28)	48.90 ± 17.65	19.23 ± 16.66	<0.001 *
Sedentary lifestyle (*n* = 43)	47.60 ± 24.83	20.94 ± 19.03	0.001 *
Another rheumatic disease (*n* = 89)	55.04 ± 21.25	23.68 ± 20.29	<0.001 *
Use of insole (*n* = 35)	52.64 ± 23.28	18.89 ± 14.39	<0.018 *

Abbreviations: BMI, body mass index; FFI, foot function index; FMS, fibromyalgia syndrome. U Mann–Whitney Test. * Statistical significance *p* < 0.05.

**Table 4:** Relationship between morphofunctional alterations in the foot and the FFI in cases and controls.

Variables		Pain	*p* Value	Disability	*p* Value	Activity Limitation	*p* Value	Total FFI Score	*p* Value
		Mean ± SD		Mean ± SD		Mean ± SD		Mean ± SD	
HV	FMS	63.94 ± 26.23	<0.001 *	58.57 ± 21.82	<0.001 *	18.50 ± 21.65	<0.001 *	51.49 ± 23.00	<0.001 *
	No FMS	27.79 ± 54.63		16.52 ± 22.79		2.16 ± 5.13		15.13 ± 17.95	
Hyperkeratosis	FMS	64.43 ± 25.60	<0.001 *	58.91 ± 28.07	<0.001 *	19.52 ± 22.85	<0.001 *	51.96 ± 23.10	<0.001 *
	No FMS	27.43 ± 55.02		15.62 ± 21.63		2.08 ± 5.11		15.23 ± 17.38	
Pronated foot	FMS	62.34 ± 28.57	<0.009 *	59.89 ± 27.37	<0.001 *	23.77 ± 30.23	0.033 *	52.55 ± 24.83	0.002 *
	No FMS	39.31 ± 30.34		26.17 ± 27.24		5.51 ± 9.46		26.24 ± 22.68	
Supinated foot	FMS	80.14 ± 10.78	<0.001 *	72.59 ± 26.50	<0.001 *	20.78 ± 23.34	0.019 *	65.39 ± 17.71	<0.001 *
	No FMS	8.9 ± 12.68		6.05 ± 9.99		1.33 ± 2.81		5.87 ± 8.02	
Limitation DF of 1st MTP Join	FMS	63.43 ± 23.35	<0.001 *	59.18 ± 26.91	<0.001 *	18.37 ± 21.95	<0.001 *	51.57 ± 22.13	<0.001 *
	No FMS	25.36 ± 52.02		15.37 ± 21.96		1.90 ± 4.86		14.52 ± 17.41	
Limitation DF of TPT joint (with knee flexed)	FMS	58.26 ± 29.80	<0.001 *	60.02 ± 32.43	<0.001 *	15.41 ± 18.01	<0.001 *	49.74 ± 26.09	<0.001 *
	No FMS	39.16 ± 102.40		12.17 ± 18.46		2.33 ± 7.22		11.31 ± 15.14	
Limitation DF of TPT joint (with knee extended)	FMS	64.09 ± 26.14	<0.001 *	58.93 ± 28.65	<0.001 *	17.47 ± 21.53	<0.001 *	51.40 ± 19.49	<0.001 *
	No FMS	27.97 ± 62.62		13.60 ± 20.30		1.98 ± 5.17		13.28 ± 16.78	
Flexor hallucis brevis active	FMS	69.61 ± 23.95	0.012 *	63.92 ± 26.81	0.019 *	20.84 ± 2.66	0.027 *	56.27 ± 21.94	0.006 *
	No FMS	45.68 ± 28.51		33.92 ± 35.15		3.89 ± 7.00		30.86 ± 23.31	
Adductor hallucis active	FMS	69.61 ± 23.95	0.012 *	63.92 ± 26.81	0.019 *	20.84 ± 23.66	0.027 *	56.27 ± 21.94	0.006 *
	No FMS	45.68 ± 28.51		33.92 ± 35.15		3.89 ± 7.00		30.86 ± 23.31	
Dorsal interosseous active	FMS	69.04 ± 24.10	0.001 *	63.15 ± 26.34	0.005 *	21.45 ± 23.19	0.010 *	56.07 ± 21.64	0.002 *
	No FMS	46.52 ± 24.37		37.70 ± 25.46		6.32 ± 9.72		33.41 ± 19.13	
Extensor digitorum brevis active	FMS	70.07 ± 22.68	0.114	66.88 ± 25.56	0.057	21.63 ± 24.66	0.229	57.55 ± 21.47	0.057
	No FMS	9.52		1.23		0.00		3.70	
Quadratus plantae active	FMS	68.12 ± 23.63	0.389	63.63 ± 28.43	0.072	20.95 ± 24.29	0.237	55.88 ± 22.39	0.089
	No FMS	59.17 ± 25.83		37.04 ± 3.64		4.44 ± 3.63		37.74 ± 17.43	

Abbreviations: 1st MTP, first metatarsophalangeal; DF, dorsiflexion; FFI, foot function index; FMS, fibromyalgia syndrome; HV, hallux valgus; TPT, tibiofibular-talar. U Mann–Whitney Test. * Statistical significance *p* < 0.05.

**Table 5:** Logistic regression to determine the factors influencing the presence of FMS.

	Beta	Standard Error	Wald	gl	Sig.	Exp(B)	95% CI for EXP(B)	
							Lower	Upper
Overweight or obesity	0.671	0.388	2.992	1	0.084	1.955	0.915	4.181
Low educational level	0.942	0.409	5.300	1	0.021 *	2.566	1.150	5.725
Current smoker	−0.278	0.530	0.275	1	0.600	0.757	0.268	2.141
Sedentary lifestyle	0.022	0.437	0.002	1	0.960	1.022	0.434	2.407
Another rheumatic disease	1.624	0.395	16.905	1	<0.001 *	5.071	2.339	10.995
Use of insole	0.670	0.521	1.653	1	0.199	1.954	0.704	5.423
Moderate or severe HV	0.621	0.417	2.220	1	0.136	1.861	0.822	4.215
Hyperkeratosis	−0.754	0.552	1.868	1	0.172	0.470	0.160	1.387
Supinated foot	−0.408	0.719	0.321	1	0.571	0.665	0.162	2.724
Pronated foot	−0.237	0.537	0.195	1	0.659	0.789	0.275	2.260
Limitation of DF of the TPT joint	0.244	0.411	0.352	1	0.553	1.276	0.570	2.856
Any active trigger point	2.412	0.527	20.982	1	<0.001 *	11.154	3.974	31.305
Constant	−1.658	0.606	7.487	1	0.006	0.191		

Dependent variable: FM diagnosis (yes/no). Abbreviations: DF, dorsiflexion;FMS, fibromyalgia syndrome; HV, hallux valgus; TPT, tibiofibular-talar. * Statistical significance *p* < 0.05.

## Data Availability

The original contributions presented in the study are included in the article, further inquiries can be directed to the corresponding authors.
